# Effect of active forced air warming during the first hour after anesthesia induction and intraoperation avoids hypothermia in elderly patients

**DOI:** 10.1186/s12871-022-01577-w

**Published:** 2022-02-07

**Authors:** Jingyu Wang, Ping Fang, Gangqiang Sun, Ming Li

**Affiliations:** 1 Department of Surgical Anesthesia Center, LiHuili Hospital, Ningbo Medical Center, No.57 Xingning Road, Yinzhou District, Ningbo, 315000 Zhejiang Province China; 2 Department of Surgical Anesthesia Center, the Second Hospital of Haishu District, No.52 Yizhi Middle Road, Shiqi Street, Haishu District, Ningbo, 315000 Zhejiang Province China

**Keywords:** Forced-air warming, Hypothermia, First hour after induction, Elderly patients, Laparoscopic abdominal surgery

## Abstract

**Background:**

The study aimed at exploring an optimal temperature model of forced air warming during the first hour after induction and intraoperation to prevent hyperthermia for elderly patients undergoing laparoscopic abdominal surgery.

**Methods:**

There were 218 patients that were randomly divided into 3 groups warmed with a forced-air warmer during surgery: Group L (intraoperative warming set to 38 °C, *n* = 63), Group H (intraoperative warming set to 42 °C, *n* = 65) and Group LH (intraoperative warming set to 42 °C for the first hour then set to 38 °C, *n =* 65). Core temperature in the preoperative room and PACU was measured by a tympanic membrane thermometer and in the operation room, a nasopharyngeal temperature probe was recorded. The rate of perioperative hypothermia, defined as a reduction in body temperature to < 36 °C was recorded as the primary outcome. Intraoperative anesthetic dosage, recovery time, adverse events, thermal comfort and satisfaction score were measured as secondary outcome.

**Results:**

The incidence of intraoperative and postoperative hypothermia was significantly lower in Group LH and Group H than Group L (18.75 and 15.62% vs 44.44%, *P*<0.001; 4.69 and 4.69% vs 20.63%, *P*<.05). Anesthetic dosage of rocuronium was lower in Group L than other two groups, with the opposite result of recovery time. The number of patients with shivering was higher in Group L but sweating was higher in Group H. Both of the thermal comfort and satisfaction score was highest in Group LH.

**Conclusion:**

A temperature pattern of forced air warming set at 42 °C during the first hour after anesthesia induction and maintained with 38 °C was a suitable choice for elderly patients undergoing laparoscopic abdominal surgery lasting for more than 120 min.

**Trial registration:**

Chictr.org.cn ChiCTR-2,100,053,211.

## Background

Intraoperative hypothermia can be caused by general anesthesia, which is defined as a core body temperature below 36 °C [1]. If suitable measures aren’t taken in time, complications such as prolonged drug metabolism, postoperative shivering, wound infections, coagulopathy and cardiac arrhythmias and increase of hospital resources [2]. In order to maintain intraoperative normothermia during anesthesia, anesthetists have insisted on exploring warming period including warming patients before induction of anesthesia or during the operation, and warming instrument such as circulating-water garments, forced-air convection or continuous infusion of warmed liquids [3]^,^ which are applied throughout the world.

Core hypothermia develops after induction of general anesthesia or peripheral nerve block because of an internal core-to-peripheral redistribution of body heat [4]. Drugs used for anesthesia such as propofol and opioids decrease vasodilatation and suppress thermoregulatory thresholds, resulting in core-to-peripheral temperature gradient [5]. This redistribution may decrease core temperature by 0.5 °C to 1.0 °C within the first hour following anesthesia and continued until the end of surgery [6], which is the main cause of adverse effect postoperatively [7]. Therefore, it’s necessary to take measures to prevent the frequency of redistribution hypothermia as well as the incidence of complications during the surgery, especially the initial hour after anesthesia.

Elderly patients particularly those who are over 65 years of age, are at high risk of hypothermia due to the less effective regulatory capacity of their central nervous system on body temperature, poor nutritional status and pre-existing diseases [8]. The guidelines recommended by the enhanced recovery after surgery (ERAS) have stated that maintaining normothermia during operation for elderly patients is necessary but overlooked in reducing the surgical complications and morbidity, particularly in abdominal surgery. Numerous factors have been associated with preoperative hypothermia such as ambient temperature [9], fluid warmers [10] and insulation measures, among which forced-air warming devices are reported to be the most commonly method during abdominal surgery [11]. However, the warming mode and duration time have been controversial according to different research previously. Some studies suggest that pre-warming reaches sufficient efficacy [12] and some support that forced-air warming system set at 42 °C is shown to be a more effective way of rewarming elderly patients [2]. Few researchers have focused on the efficacy of first hour warming after the induction of anesthesia.

The aim of our study was to evaluate the importance of intraoperative warming using forced-air warming devices with suitable temperature and feasible duration time at first hour after induction of anesthesia. We conducted this trial to search for the most effective temperature for perioperative rewarming for elderly patients undergoing laparoscopic abdominal surgery.

## Materials and methods

### Patients

This trial was approved by institutional Ethics Committee of Ningbo Medical Center Lihuili Hospital (2017024) and retrospectively registered at chictr.org (ChiCTR-2,100,053,211) on 15/11/2021. The study enrolled 218 patients scheduled for elective major laparoscopic surgery (liver, gastric and colorectal surgery) under general anesthesia with an expected operating time of at least 2 h from May 2019 to October 2020. All participating patients received written consent and information regarding the trial. The criteria for inclusion were elderly patients (age ≥ 65 years), American Society of Anesthesiology (ASA) Grades I to II, scheduled for elective laparoscopic resection of abdominal carcinoma surgery under general anesthesia for at least 2 h, and no clinical disease of nasopharynx. We excluded the patients who were obese (body mass index (BMI)) > 30 kg/m^2^, pre-existing preoperative hypothermia (35.5 °C) or hyperthermia (37.5 °C), an evidence of current infection, thyroid disease, postoperative delirium, and those who were taking drugs acted on thermoregulation such as vasodilator/vasoconstrictor medications.

A letter was generated by a computer-generated randomization table and the result was placed in a sealed envelope. Letter “L” was on behalf of Group L (intraoperative warming set to 38 °C, *n* = 63), letter “H” was for Group H (intraoperative warming set to 42 °C, *n* = 65) and letter “LH” was for Group LH (intraoperative warming set to 42 °C for the first hour then set to 38 °C until sending to PACU, *n =* 65). On the day of surgery, the nurse in the pre-operation room opened the sealed envelope and randomly allocated the patient to the intervention or control group. All of the anaesthetists, OR nurses and PACU nurses nor participants were not aware of grouping allocations.

### Anesthesia

No premedication was given to patients before surgery. Firstly, patients were transferred to the perioperative preparation room controlled to 23 °C and warmed with a regular blanket. A tympanic membrane thermistor (First TempR Genius Model 3000A, Covidien Ireland Limited Corp, Tullamore, Massachusetts) was measured for core body temperature to avoid discomfortable feelings in perioperative room to exclude hypothermia and hyperthermia patients. Then a radialartery and a puncture on the external jugular vein were inserted into the patient 20 min before sending to the operative room. Immediately after arrival in the operating room, tympanic membrane temperature was measured and recorded by nurses as “baseline”. The room temperature was kept at 22 to 24 °C with relative humidity at 40 to 60%. When the anesthesiologists were ready for the induction, the forced-air warming (Warm Touch 6000, Covidien, Mansfield, MA) covered from neck to foot was initiated by nurse under the patient’s surgical drape. Those who were assigned to Group L,the resistive warming system was set to 38 °C. For Group H, the resistive warming system was set to 42 °C, and for Group LH, the temperature was set to 42 °C for the first hour after induction and then turned to 38 °C before sending to PACU.

Anesthesia induction was provided with intravenous administration of propofol 2-2.5 mg/kg, sufentanil 0.2-0.3 mg/kg, rocuronium 0.6 mg/kg and anesthesia maintenance was done with sevoflurane (0.7-1.0 minimum alveolar concentration) and remifentanil (0.1-0.15μg/kg/min). Rocuronium (0.15 mg/kg) was infused to maintain muscle relaxation according to train-of -four (TOF) ratio [13]. An automated monitor system (Philips MP60) with electrocardiogram, invasive blood pressure, peripheral oxygen saturation and electrodes of the bispectral index (BIS, Aspect Medical System, Newton, MA, USA) were detected. A nasopharyngeal temperature probe wasinserted 10 cm and connected to the anesthesia machine immediately after induction of anesthesia. All of the intravenous and irrigation fluids were heated to 38°C by incubator (MIR-162, SANYO, Japan) during surgery. The heating temperature was switched off when the core body temperature was > 37.5 °C or when the patients became sweating. After the surgery, patient recovery was assessed by a Steward scale [14], and the nasopharyngeal thermometer and tracheal tube were removed when the consciousness and spontaneous respiration had been restored. The patients were then transferred to PACU with a blanket prewarmed to 38 °C.

### Outcome measures

The primary outcome was the incidence of perioperative hypothermia (core temperature < 36.0 °C). Each patient’s tympanic temperature was measured by a nurse in the preoperative room, immediately after arriving in the operative room and PACU at 15-min intervals until transferred to inpatient ward. A nasopharyngeal temperature was measured immediately after induction of anesthesia as “0 min” and recorded at 30-min intervals throughout the surgery. The severity of hypothermia was classified into three groups: mild hypothermia (35.5°C-35.9°C), moderate hypothermia (35.0°C-35.4°C) and severe hypothermia (< 35.0°C).

The secondary outcomes were anaesthetic drugs used (propofol, sufentail, rocuronium, remifentail and sevoflurane), time to recovery, adverse effects (such as arrhythmia, hypotension, hypertension, shivering, hypoxemia and sweating, patinets’ satisfaction score, postoperative thermol comfort score. Patients were tested by a nurse of the thermal comfort scale using an 11-point Likert scale: 0 = entirely cold, 10 = fully hot. Simiarly, patients were asked to satisfaction scale using an 11-point Likert scale:0 = entirely unsatisfied, 10 = fully satisfied. The adverse effects (such as arrhythmia, hypotension, hypertension and sweating) were recored from the induction, with the remaining effects (shivering and hypoxemia) recorded after arrivaling in the PACU.

### Statistical analysis

Our previous preliminary experiment showed that the incidence of hypothemia was 12.6% in patients who warmed with forced air warming at 38 °C at first hour and rose to 42°C thereafter, whereas it was 11.3% in patients warmed at 42°C and 36.3% warmed at 38°C respectively.

Assuming study power of 80% (a = 0.05, b = 0.2), the required sample size per group was calculated to be 58 (PASS 11.0, NCSS Statistical Software, Kaysville, Utah). For a dropout rate of 10%, the final sample size was determined to be 63 patients for each group.

Statistical analysis was performed with SPSS (version 23.0, Chicago,IL,USA). Quantitative distributed data were expressed as means and standard deviations and homogeneity of variance was determined by using Levene tests. The Kolmogorov-Smirnov test was used to assess distribution of variables. Student-Newman-Keuls was applied to compare differences between any two samples for the data with equal variance. Otherwise, Kruskal Wallis H test with the Bonferroni method was utilized to correct the significance level for post-hoc multiple comparisons. Categorical data was expressed as number (n) and percentage (%) using chi-square tests or Fisher exact tests. Probability (*P*) values <.05 were considered statistically significant.

## Results

### Baseline characteristics

**Fig. 1:**
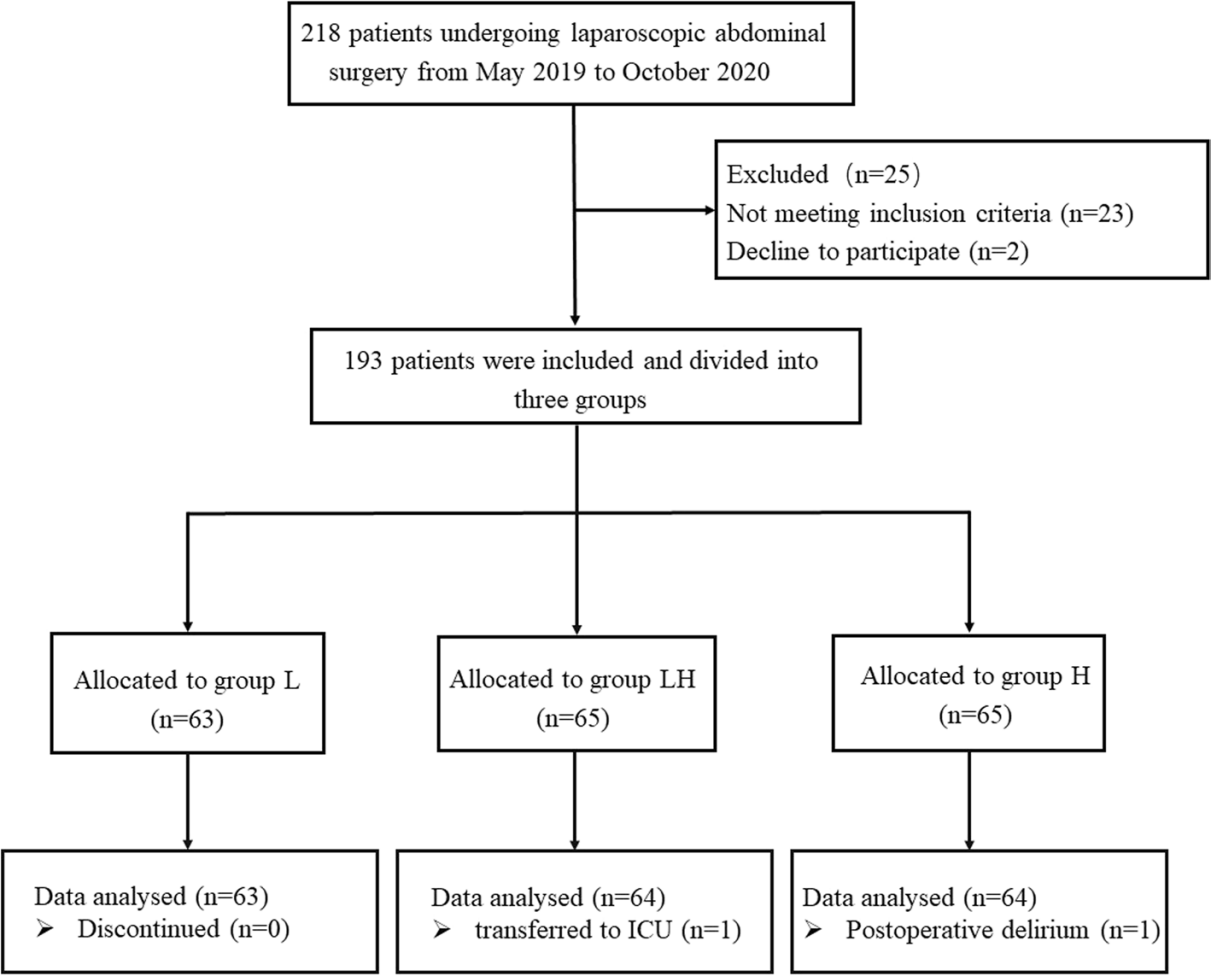
Flow diagram of pateint enrollment

The diagram of patients enrollment in this study is shown in Fig. 1. There were 218 patients.

that were screened for research in total but 25 patients who did not meet the inclusion criterias were excluded and 2 patients declined to participate. Therefore, only 193 patients ultimately enrolled and divided randomly into the Group L (intraoperative warming set to 38°C, *n* = 63), Group H (intraoperative warming set to 42°C, *n* = 65) and Group LH (intraoperative warming set to 42°C for the first hour then set to 38°C until transfering to PACU, *n =* 65). Of those, patients transferred to the intensive care unit in Group LH(*n* = 1) and showed postoperative delirium in the PACU in Group H (*n =* 1). Data obtained from these patients could not be assessed. Finally, there were 191 patients were available for analysis: Group L(*n* = 63), Group LH (*n* = 64) and Group H (*n =* 64).

There were no difference between the baseline characteristics of the patients (age, sex, weight, BMI, and ASA physical status classification). The following surgical characteristics such as initial body temperature, operating room temperature, PACU temperature, surgery type, time of anesthesia incubation, surgery duration, anesthesia duration, fluid administration volume and blood loss made no significant sense in three groups identically (*P*>.05, Table 1).

**Table 1:**
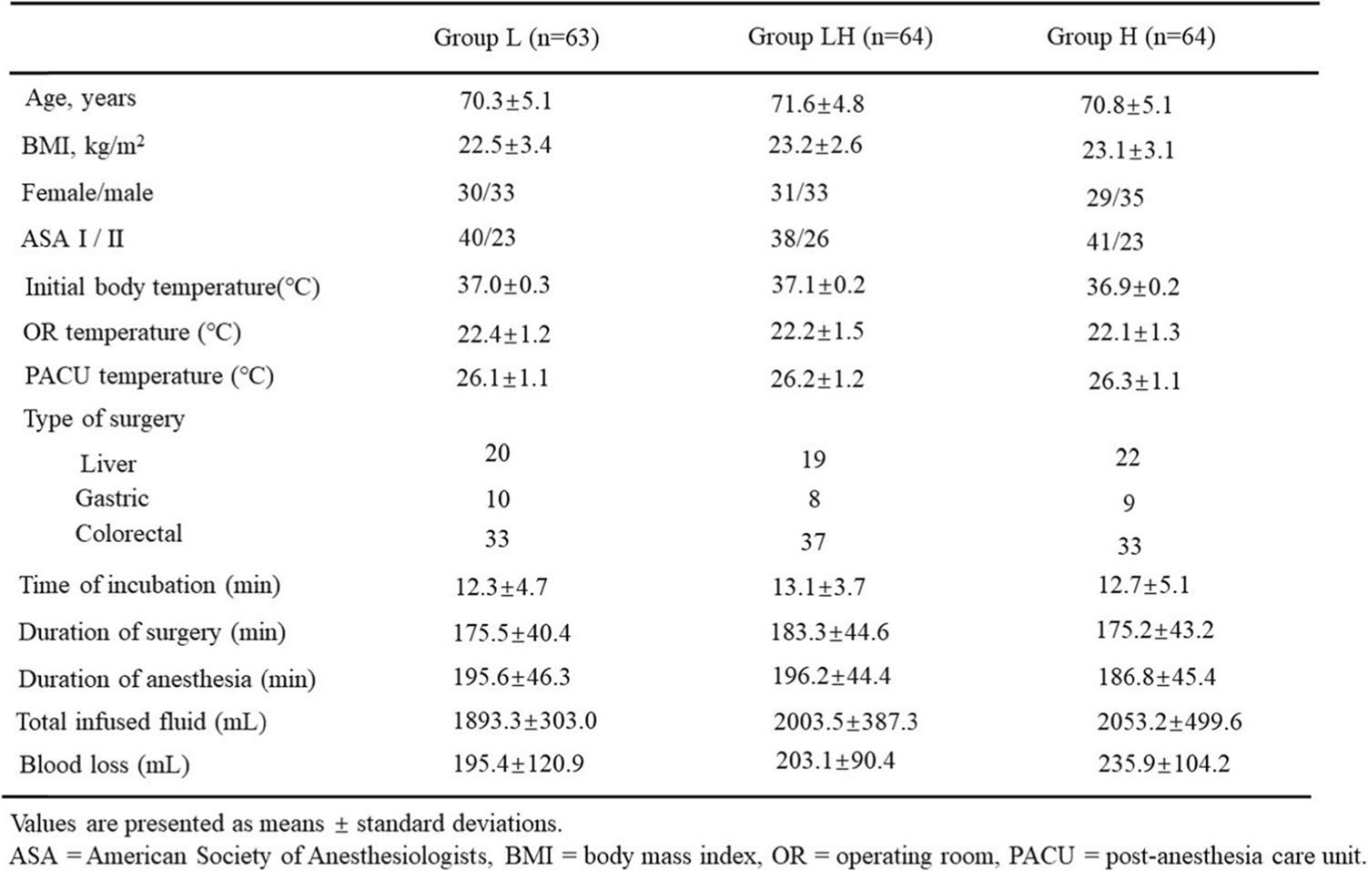
Patients’ characteristic and Intraoperative data. Values are presented as mean ± standard deviations. ASA = American Society of Anesthesiologists, BMI = body mass index, OR - operating room, PACU = post-anesthesia care unit.

### Perioperative hypothermia data

The incidence of intraoperative hypothermia was statistically different in Group L, GroupLH and Group H (44.44% vs 18.75 and 15.62%, resperctively, *P*<.001, Table 2). Compared with Group LH and Group H, the rate of intraoperative hypothermia in Group L was significently higher.

**Table 2:**
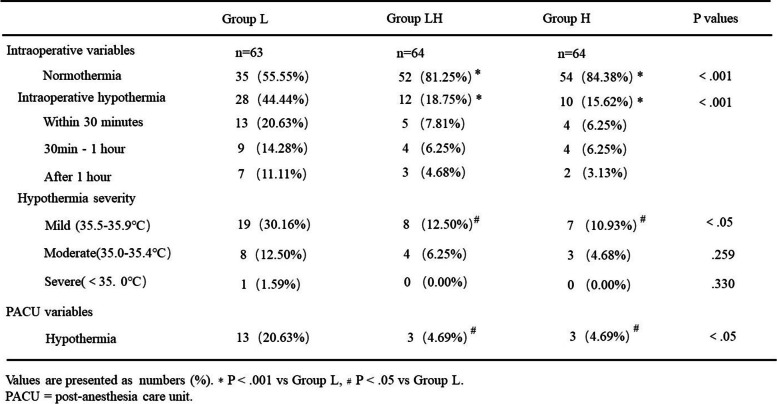
Intraoperative and postoperative variable. Values are presented as numbers (%). * *P* < .001 vs Group L, * *P* < .03 vs Group L. PACU = post-anesthesia care unit.

There was no meaningful diference between Group LH and Group H (*P*>.05). As the results shown, more than half of the patients had been hypothermic after anesthesia induction within one hour, which existed in all of the three groups, indicating that anesthesia induction had a severe impact for the first-hour-onset hypothermia for elderly patients during surgery. Compared with Group LH and Group H, Group L had got a higher rate of mild degree of hypothermia(30.16% vs 12.50 and 10.93%, *P*<.05), but made no sense in moderate and severe degree. Likewise, the patients of Group L in PACU were more liable to be hypothermic than the other two groups (20.63% vs 4.69 and 4.69%, resperctively, *P*<.05), similar to the outcomes intraopertively.

Neither the rate of mild hypothermia or hypothermic in PACU was comparable in Group LH and Group H (*P*>.05).

**Fig. 2:**
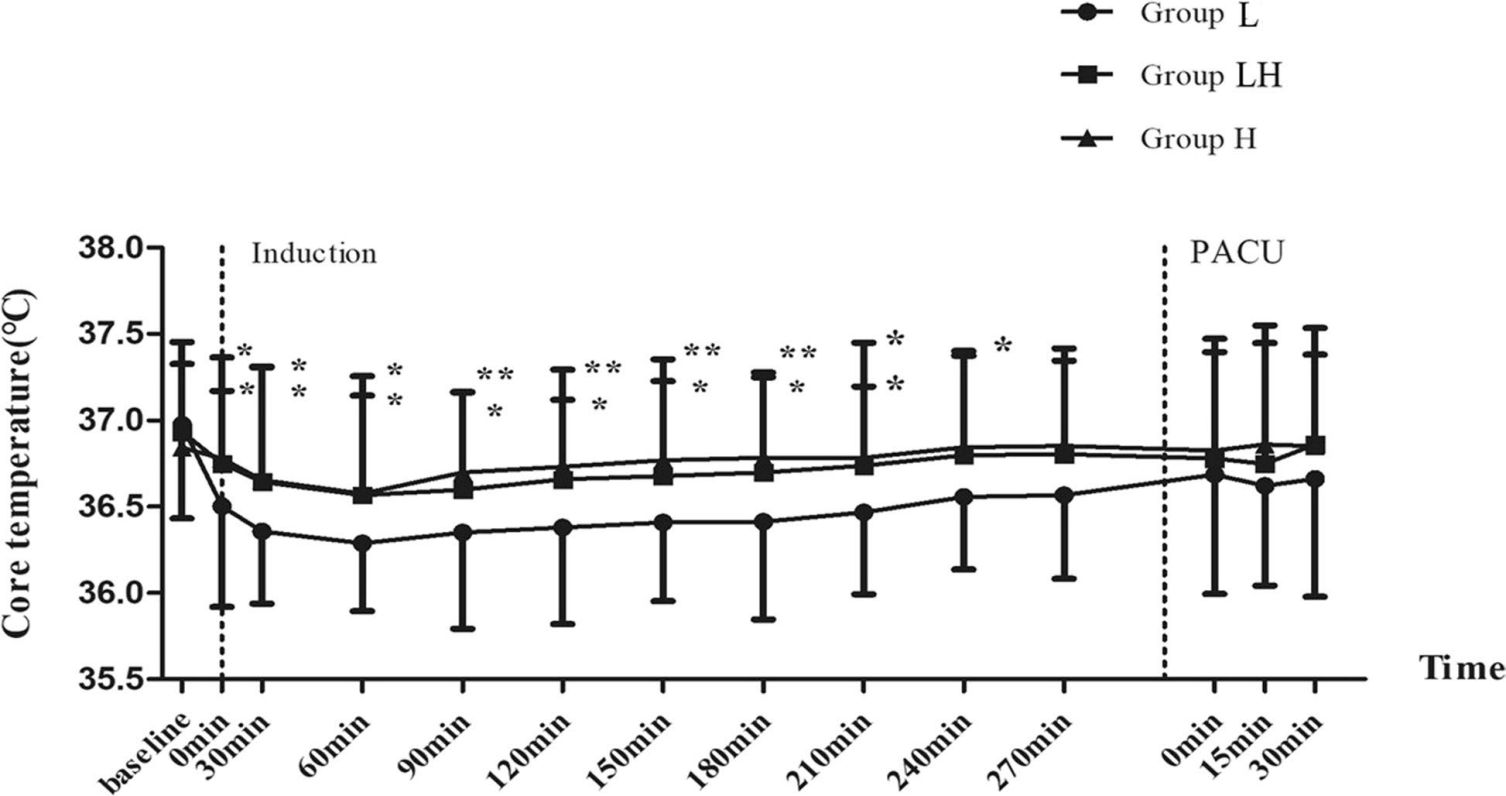
Perioperative body temperature. Preoperative and postperative core temperature of patients were measured ussinng tympanic membrane thermometer. Intraoperative core temperature was recorded at 30-min intervals via nasopharyngeal probe after induction of anesthesia. Baseline: immediately after arrival in operation room; intraoperative 0 min: immediately after insertion of nasopharyngeal probe; PACU 0 min: immediately after arrival at PACU; PACU 15, and 30 min: 15 and 30 min after arrival at PACU. * represents *P<*, 05 compared with Group L, ** represents *P <* .001 compared with Group L.

The core temperature change between groups over time were shown in Fig. 2. The temperature of Group L was lower than Group LH and Group H from induction of anesthesia within one hour (*P*<.05). Thereafter, the difference between Group L with Group LH (*P*<.05) and Group L with Group H (*P*<.001) was expanded until 210 min after induction. Up to 240 min, the temperature between three groups were homogenous since 270 min after induction until leaving PACU (*P*>.05, Fig. 2). However, the results between Group LH and Group H were homologous.

(*P*>.05).

### Warming effects in intraoperative period and adverse events

Warming effects on anesthesia were shown in Table 3. Anaesthetic drugs such as propofol, sufentanil, remifentail and sevoflurane were comparable among three groups. However, the usage of rocuronium had shown distinction among Group L, Group LH and Group H (56.36 ± 5.02 vs 64.29 ± 5.21 and 64.25 ± 5.12 resperctively, *P*<.001, Table 3), with lower usage in Group Lthan the other two groups. The results between Group LH and Group H were comparable (*P*>.05). On the contrary, time to recovery was higher in Group L than the other two groups (33.03 ± 4.53 min vs 26.75 ± 3.27 min and 25.60 ± 2.82, *P*<.001, Table 3) and the difference between Group LH and Group H was still comparable (*P*>.05). Besides, compared to Group LH and Group H, those patients in Group L had a higher incidence of shivering (39.68% vs 17.19 and 18.75%, *P*<.05). Nevertheless, the rate of sweating was higher in Group H than Group LH (9.38% vs 3.13%, *P*<.05), with no one happened in Group L. No difference was respected among the three groups with the incidence of arrhythmia, hypotension, hypertension and hypoxemia.

**Table 3:**
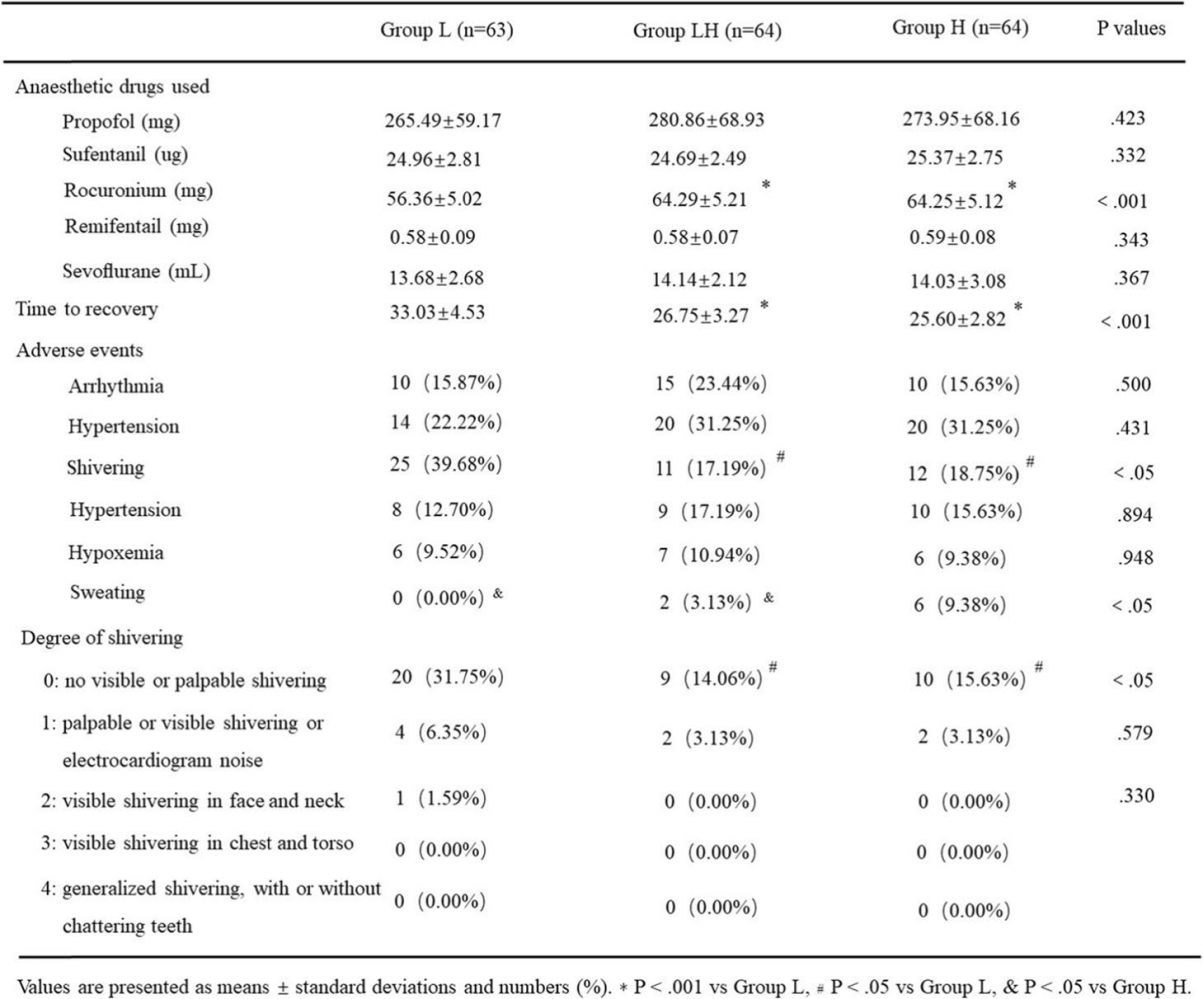
Comparison of warming ± effects in perioperative period. Values are presented as means standard deviations and numbers (%). * *P*<. 001 vs Group L, * P<. 05 vs Group L, & P<. 05 vs Group H.

Furthermore, we analysed the shivering grades in three groups. The number of patients with a shivering grade of 0 were significantly higher in Group L than the other two group (31.75% vs 14.06 and 15.63%, *P*<.05), while no statistic change were found in other grade (*P*>.05, Table 3). Neither of the two gaps for adverse events had shown difference in Group LH and Group H (*P*>.05). Both of thermal comfort and patients’ satisfaction score reached the highest level in Group LH than Group L (*P*<.001) and Group H (*P*<.05). There was no obvious difference between Group L and Group H (*P*>.05).

## Discussion

This study demonstrated that both the intraoperative warming set to 42 °C for the first hour then set to 38 °C group and intraoperative warming set to 42 °C group reduced the incidence and severity of inadvertent perioperative hypothermia compared to the intraoperative warming set to 38 °C group in elderly patients undergoing major abdominal surgery lasting more than 120 min. Additionaly, higher incidence of perioperative hypothermia was associated with lower usage of anaesthetic drugs but longer recovery time. Though all of the groups got same level of core temperature at the end of surgery, the intraoperative warming set to 42 °C for the first hour then set to 38 °C group achieved highest thermal comfort and satisfaction score in PACU.

Patients with abdominal surgery are at high risk of hypothemia because of the prolonged exposure of large surface of skin to cold temperatures and durtion of long-lasting operative time, especially under genernal anesthesia. Currently, as many as 52.7% of general surgery patients undergoing abdominal operations have a temperature <36 °C from the start of operation and the incidence increased sustainably until the end of surgery if appropriate heating meaures were not taken [15]. Active rewarming strategies are sincerely necessary for hypothermic patients, which are applied during perioperation, intraoperation and postoperation. Forbes et al reported that pre-warming at least 30 min could reduce the incidence of hypothemia and postoperative complications [16]. Contrastly, Yoo *at el* suggested that even warming during induction of anesthesia had sufficient efficacy to prevent hypothermia and reduces shivering [17]. Both of the studies had stated the advantange of preventing hyperthermic during the first-onset of anesthesia induction. However, Su et al found that no difference was existed from induction of anaesthesia until 30 min after starting of operation but dominant superiority appeared from 60 to 240 min into surgery [18]. Furthermore, Zhang et al rewarmed patients in PACU, which had the similar warming effect compared to the pre-warming reasearch [19]^.^ In brief, forced-air warming system was an simple and convenient method to prevent inadvertent perioperative hypothermia, but a more effective way and suitable temperature should be explored.

Based on the previous studies, we warmed patients using the forced-air warming system at 42°C for the first hour from anesthesia induction, and sustained the temperature for at least one hour with 38°C or 42°C in Group LH and Group H individually. Besides, Group L was warmed with 38°C throughout the surgery. Most of the patients had been hypothermic within one hour after induction, and the reduced core temperature in Group L was approximately 0.6°C, which were comparable to the studies employed with pre-warming methods [20, 21]. The decrement in Group L and Group H was 0.3°C, indicating that 42°C had better heat preservation for the first hour. Thereafter, the difference of reduced temperature was expanded between Group L and Group H compared to Group L and Group H in the next 210 min, and gradually dispeared till 270 min and in PACU. There was no statically difference since the first hour of induction no matter waremed with 38°C or 42°C, which were consisent with the previous researches [8]. It was the first study for us to indicate that warmed at 42°C rather than 38°C with forced-air warming system was necessary for the reduction of temperature caused by anesthesia induction during the first hour but the impact was shrinked as the surgery were sustained .

Mild hypothermia may result in prolonged recovery from anesthesia because of delayed metabolism and clearance of anesthetic drugs. Bjelland et al has reported that therapeutic hypothermia could change the concentrations of remifentanil, propofol, and midazolam either during cooling and rewarming time, with no adjustment for fentanil [22]. Leslie et al has explained that reduced hepatic blood flow altered the volumes of distribution of propofol by decreased 3°C of core hypothermia. Meanwhile the durtion of atracurium was 60% prolonged in the same hypothemia degree [23]. Consistent with those results, Lee et al has proved that rocuronium recovery was prolonged by even lower hypothermia (nasopharyngeal temperature:30.4°C) [24].

In our study, we found that the usage of propofol, sevoflurane, sufentanil and remifentail were homologous among the there groups. Nevertheless, the consumption of rocuronium was lower in Group L than in Group LH and Group H. On the contrary, the time to recovery was highest in Group L, suggesting that the elimination of muscle relaxant may be influenced by hypothermia during surgery with the temperature higher than other studies [24, 25]. Besides, in our study, the dosage of sedatives and analgesics were similar due to the normal range of BIS, which was comparable to the previous study that core temperature (between 35°C and 38°C) had no sigfinicant influence on BIS values [26].

Hypothemia weakened the thermal retardation capability during surgery, especially among those elderly patients, develping with cardiac complications, shivering, hypoxemia and sweating.

The reason accounted for the high risk of cardiac complications was the consequential increase in plasma norepinephrine concentrations, which promoted vasoconstriction with consequent hypertension and development of tachycardia. In our study, the incidence of arrhythmia, hypertension, hypotension,hypoxemia except sivering and sweating showed no difference between the three groups, which was accordance with the results in previous studies [2, 18]. However, the disparity of shivering severity between Group L and Group LH or Group H existed in grade 0 merely. This was probably due to the fact that the basic temperature in our study was 38 °C rather than normalthermic and no premedication was used in other studies. For thermal comfort and patients’ satisfaction score, Group LH reached a higher level than Group L and Group H, as the rate of sweating was rised significantly in Group H. The above results demostrated that set the temperature at 38 °C was equal to 42 °C in heat preservation intraoperatively but achieved a more preferable satisfaction postoperatively.

This study has some limitations here. Firstly, we only included elderly patients more than 65-year-old, who had a higher risk of preoperative hypothemia actually, which could increase the incidence of hypothemia especially having the abdominal surgery. Secondly, we used tympanic thermometer and nasopharyngeal temperature in preoperative room and operative room respectively, which may effect the accurancy of results. Thirdly, we only recorded the adverse effects in PACU but no information in inpatient ward. As a result, we couldn’t predict the long-lasting effect of patients caused by hypothemia.

## Conclusions

In summary, we recommended a new warming therapy that set the forced-air warming system at 42 °C within the first hour, then turned the temperature to 38 °C, could be an effective, convenient and satisfying method to deal with the preoperative hypothemia happened in the abdominal surgery lasting for more than 2 h.

## Data Availability

The anonymized dataset will be available by reasonable request sent to the corresponding author.

## Abbreviations

ASA: American Society of Anesthesiologists
BMI: Body mass index
PACU: post-anesthesia care unit
